# Video-rate volumetric neuronal imaging using 3D targeted illumination

**DOI:** 10.1038/s41598-018-26240-8

**Published:** 2018-05-21

**Authors:** Sheng Xiao, Hua-an Tseng, Howard Gritton, Xue Han, Jerome Mertz

**Affiliations:** 1 Department of Electrical & Computer Engineering, Boston University, 8 Saint Mary’s St., Boston, Massachusetts 02215 USA; 2 Department of Biomedical Engineering, Boston University, 44 Cummington Mall, Boston, Massachusetts 02215 USA

## Abstract

Fast volumetric microscopy is required to monitor large-scale neural ensembles with high spatio-temporal resolution. Widefield fluorescence microscopy can image large 2D fields of view at high resolution and speed while remaining simple and costeffective. A focal sweep add-on can further extend the capacity of widefield microscopy by enabling extended-depth-of-field (EDOF) imaging, but suffers from an inability to reject out-of-focus fluorescence background. Here, by using a digital micromirror device to target only in-focus sample features, we perform EDOF imaging with greatly enhanced contrast and signal-to-noise ratio, while reducing the light dosage delivered to the sample. Image quality is further improved by the application of a robust deconvolution algorithm. We demonstrate the advantages of our technique for *in vivo* calcium imaging in the mouse brain.

## Introduction

Recording neuronal function at relevant spatial and temporal scales is a crucial step towards understanding brain function^1^. The advent of genetically encoded optical indicators has facilitated such recording with a light microscope^2^. However, interconnected neurons can often extend over hundreds of micrometers and indicator response times are often on the order of tens of milliseconds. An imaging system capable of monitoring extended volumes at high speeds is thus required^1^. In general, point scanning techniques such as confocal^3^ or two-photon microscopy^4^ are too slow for real-time volumetric imaging applications, though efforts have been made to increase their temporal resolution using, for example, random access scanning^5–7^, point spread function (PSF) engineering^8,9^, or spatial/temporal multiplexing^10–12^. Widefield-based techniques, on the other hand, can benefit from high-sensitivity cameras with massive pixel counts^13^ that can capture wide 2D fields of view (FOVs) within a single snapshot. This is particularly advantageous for volumetric imaging since it obviates the need for 2D scanning, offering the potential for increased imaging speed and reduced system complexity. For example, light sheet microscopy with fast single-axis scanning can achieve video-rate volume imaging in the brain^14,15^, though the requirement of side-on illumination often makes it impractical, prescribing in some cases oblique light sheet delivery^16^.

Of all the techniques available, conventional widefield fluorescence microscopy remains one of the most attractive for imaging surface layers or weakly scattering samples because of its high spatial and temporal resolution, high throughput, low cost and simplicity. But the tradeoff between lateral resolution and depth-of-field (DOF) still limits its capacity for volumetric imaging. Various extended depth-of-field (EDOF) approaches have been developed to counter this problem, such as wavefront coding^17,18^ or spectral/spatial multiplexing^19,20^, or making use of diffractive/refractive elements such as an axicons or fresnel zone plates^21,22^.

Yet another way to perform EDOF imaging is by sweeping the microscope focal plane within the camera exposure time. Such sweeping can be achieved by physically translating the sample^23^ or sensor^24^, or alternatively by remotely scanning the microscope focus with a remote mirror^25^, electrically tunable lens (ETL)^26–29^, tunable acoustic gradient lens^30^, or deformable mirror^31,32^. All of these remote focus scanning strategies are sufficiently fast that they can provide video-rate EDOF imaging. However, as is the case with most widefield-based techniques, they do not reject out-of-focus background. Specifically, objects in an EDOF image are both in focus and out of focus. The out-of-focus contributions constitute background haze that degrades both the image contrast and signal-to-noise ratio (SNR), which will be exacerbated with longer DOF extension. Although this problem of background haze can be partially alleviated by deconvolution^32^, the shot noise associated with a large background combined with the finite dynamic range of the camera ultimately limit the final EDOF image quality as well as the imaging DOF.

In this paper, we describe a widefield-based imaging technique that is capable of imaging extended volumes at video-rate with significantly improved contrast and SNR compared to conventional focal sweep techniques. The basic idea is to generate an EDOF image using 3D spatially targeted illumination (TI). Specifically, during the focal sweep, at each focal plane, instead of using uniform illumination (UI) we use patterned illumination that targets only in-focus sample structure. We do this by pre-determining the overall 3D illumination pattern that coincides with the sample structure within the scan volume. Throughout this paper, we concentrate on functional neuronal imaging, which we believe is an important application of this technique. Depending on the sparsity of neuronal labeling, our 3D illumination patterns become commensurately sparse, thus reducing the light dose inflicted on the sample. And because the illumination patterns are tailored to the sample, this reduction in light dose leads to a reduction only of the out-of-focus background and not of the in-focus signal, thus leading to an increase in contrast and SNR. Additionally, we take advantage of an adapted deconvolution algorithm to further enhance contrast and SNR, resulting in EDOF image qualities that approach those obtained by standard confocal microscopy, at 3D-volume acquisition rates orders of magnitude faster. The only requirement of our technique is that the sample be spatially fixed, though it can be temporally dynamic. This requirement can be realized in many applications. In particular, we demonstrate the advantages on our TI-EDOF technique for *in vivo* calcium imaging in head-fixed mouse brains. To our knowledge, EDOF imaging with a conventional widefield microscope has not been applied before to fast functional volumetric imaging in rodent brains, most likely because it provides insufficient contrast and SNR. We show here that the addition of TI to EDOF largely resolves this problem.

## Principle

**Figure 1:**
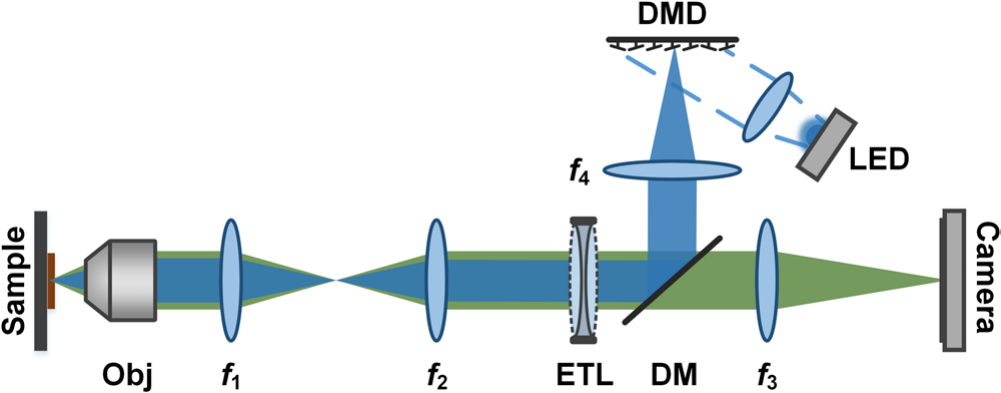
Experimental setup. *f*_1_ = 150 mm, *f*_2_ = 100 mm, *f*_3_ = 150 mm, *f*_4_ = 125 mm. Obj: objective. DM: dichromatic mirror. Additional excitation and emission filters are not shown.

Our experimental setup is shown in Fig. 1. The system is analogous to a conventional epi-fluorescence microscope in that an objective (Olympus LUCPLFLN 20×/0.45) and tube lens *f*_1_ image the sample onto an intermediate image plane, and an additional 4f system *f*_2_ and *f*_3_ relays the intermediate image onto a camera (Andor Zyla 4.2). However, there are two key additions to our system: a fast focal sweep mechanism and a targeted illumination system.

In our case, the focal sweep mechanism is an ETL (Optotune VIS-EL-10-30-C), which is inserted in a pupil plane common to both the excitation and emission beams to ensure system telecentricity. The focus shift produced by the ETL is given by

1 $$D=-\,n\frac{{f}_{obj}^{2}}{{f}_{1}^{2}}\frac{{f}_{2}^{2}}{{f}_{ETL}}$$

where *n* is the refractive index of the sample, and *f*_*ETL*_ is the focal length of ETL^14^. For example, in the case of mouse brain (*n* = 1.35^33^) and with an ETL focal length that varies from from −666 mm to infinity to +286 mm, we can attain an EDOF range of 240 μm.

Our targeted illumination system is a digital micromirror device (DMD–Texas Instruments V-7000 VIS), which is capable of projecting high resolution (1024 × 768) excitation patterns at high switching speeds up to 22.7 kHz. The DMD is placed in a plane conjugate to the sample, and projects patterns onto the sample that are defined by the “on” pixels of the DMD. A LED (Thorlabs M470L3, 470 nm) provides collimated illumination that is incident at a 24° angle relative to the DMD surface normal. To ensure that the incident and reflected illumination beams are in the same horizontal plane, the DMD is rotated 45° about its axis. To ensure that camera and DMD pixels align, the camera is correspondingly also rotated 45°.

**Figure 2:**
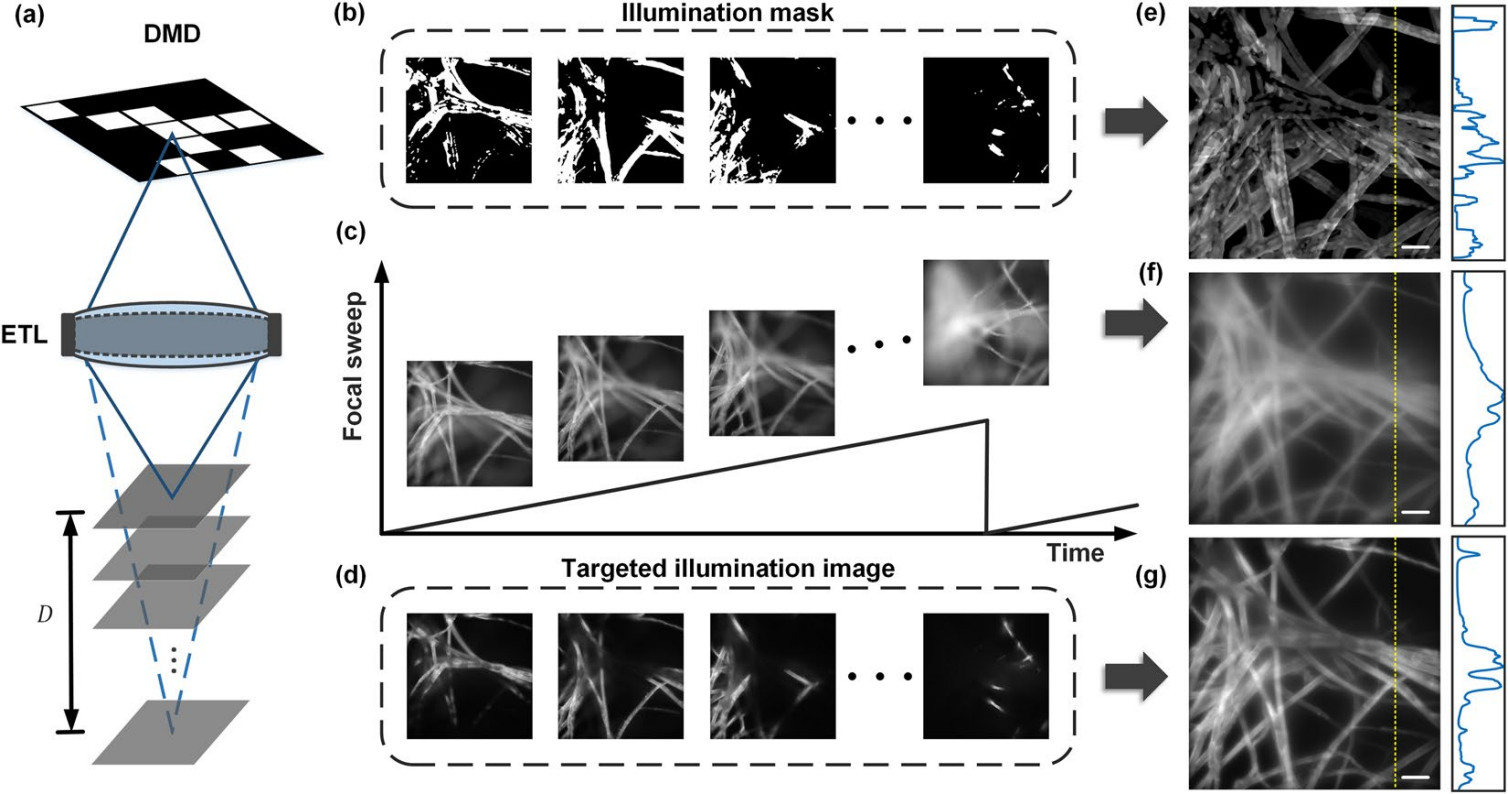
(**a**) Schematic of our method. (**c**) Images of fluorescent tissue paper acquired at different depths with UI. (**d**) Images of the same sample acquired with TI at the same depths. The associated TI patterns are shown in (**b**). (**e**) is the summation of all the TI patterns from (**e**) over a full focal scan. (**f**) is single shot EDOF image obtained with uniform illumination. (**g**) is the single-shot EDOF image obtained with TI using the binary patterns shown in (**b**). Plots depict the intensity profiles along the yellow dashed line in each image. Scale bar, 50 μm.

Combining the above two systems, we are able to target illumination at arbitrary locations within the 3D sample volume. The operating principle of our system is depicted in Fig. 2(a). While the DMD can project only transverse 2D patterns, the ETL can scan this pattern axially. By rapidly switching the DMD patterns during the ETL scan, we can focus different illumination patterns onto different axial planes, thus effectively creating a user-defined 3D illumination structure. All this is done within a single camera exposure time, enabling us to capture single-shot TI-EDOF images.

To illustrate this operating principle, we imaged fluorescent tissue paper. Figure 2(c) shows images of the sample obtained at four different focal depths using conventional UI over an axial range of 140 μm. If instead of UI we use illumination patterns (Fig. 2(b)) that target only the in-focus sample structures at each focal depth, we obtain the images shown in Fig. 2(d). Manifestly, these exhibit lower background and higher contrast than the conventional UI images. Moreover, we can produce EDOF images with UI and TI by scanning through the same 140 μm focus range within a single camera exposure, as shown in Fig. 2(f,g). We observe that the TI-EDOF image still retains the sample structural information but with significantly reduced background haze.

Of course, the operating principle described above is based on the critical assumption that the correct TI patterns are known a priori. In other words, before TI-EDOF imaging can be performed, the TI patterns must be determined in advance. In our case, we determine these patterns using structured illumination microscopy (SIM), or rather a variant of SIM similar to the technique described in^34^. The details of our approach are described in Supplementary Information. In brief, at every focal depth, we use SIM to produce an optically sectioned image of the sample structure that is in focus at that depth. This allows us to define a binary illumination pattern that specifically targets only the in-focus sample features. This procedure is repeated at every sample depth within our EDOF scan range, thus allowing us to compile an array of illumination patterns specific to each depth. Once this array is compiled and stored in computer memory, we can then perform fast TI-EDOF as described above, using the maximum DMD update rate of 22.7 kHz per pattern.

A disadvantage of SIM is that a sequence of images is required to produce a final optically-sectioned image, thus undermining its speed. However in our case, SIM is used only in the calibration step, to determine the illumination patterns subsequently required for TI-EDOF. Because speed is generally not critical for this calibration step, we are allowed a degree of flexibility in our choice of SIM illumination structures. In our case, we use a sequence of checkerboard-like structures of user-defined grid pitch and sparsity (fill factor). We can easily adapt these checkerboard features to the properties of our sample. For example, if the sample is dense or highly scattering, it is better to use a larger grid pitch and/or increased sparsity so as to ensure that the illumination structures remain resolvable. Increased illumination sparsity also has the benefit of reducing background (and hence background-associated shot noise), thus providing increased dynamic range to allow more accurate identification of in-focus signal. Although increased sparsity also means that more SIM illumination structures are required to obtain full coverage of the FOV, this only affects the speed of the calibration step and not the speed of TI-EDOF imaging itself.

Once the TI-EDOF images are acquired, these can be further improved by deconvolution. In particular, we use an algorithm borrowed from conventional UI-EDOF. Because the detection PSF is almost independent of depth within the focal scan range, one can deconvolve with a single 2D PSF, or EPSF, to remove blurring and improve SNR of UI-EDOF images^24^. Previously, we examined the associated depth-invariant 2D OTF (EOTF) for such imaging, and found this to be approximated by

2 $${\rm{EOTF}}({{\kappa }}_{\perp };D)=\,{\rm{\min }}\{\begin{array}{l}\frac{2}{\pi }({\cos }^{-1}\eta -\eta \sqrt{1-{\eta }^{2}})\\ \frac{4\kappa }{\pi D{\rm{\Delta }}{\kappa }_{\perp }^{2}\eta }\sqrt{1-{\eta }^{2}}\end{array}$$

where *κ*_⊥_ = (*κ*_*x*_, *κ*_*y*_) is the transverse spatial frequency, *κ* = *n*/*λ* is the wavenumber, Δ*κ*_⊥_ = 2*NA*/*λ* is the system bandwidth, *κ*_⊥_ = |*κ*_⊥_| ≤ Δ*κ*_⊥_, *η* = *κ*_⊥_/Δ*κ*_⊥_, *λ* is the wavelength, and NA is the numerical aperture of the objective^32^. This approximation is valid for arbitrary focal scan range *D*. To avoid overamplifying noise, we implement deconvolution as a Wiener-Helstorm (WH) filter, defined by

3 $${W}_{WH}({{\kappa }}_{\perp };D)=\frac{{\rm{EOTF}}{({{\kappa }}_{\perp };D)}^{\ast }}{|{\rm{EOTF}}({{\kappa }}_{\perp };D{)|}^{2}+\varepsilon }$$

where *ε* is the regularization parameter^35^. We set *ε* = 0.01 for all the experiments that follow. One must be careful, though. Because the illumination is spatially variant when performing TI-EDOF imaging, the 3D PSF is also spatially variant. In principle, we can calculate the exact 3D PSF for each spatial location based on our knowledge of the applied TI patterns, however such a strategy would be computationally intensive. Instead, we rely on the fact that 2D EDOF deconvolution appears to be quite robust and insensitive to PSF discrepancies^35^. In the case of TI-EDOF, we have found that a single 2D EPSF for deconvolution still yields excellent results, with the scan parameter *D* in Eq. 2 chosen to be smaller than the full EDOF scan range (more on this below).

## Results

### Contrast and noise

As described above, our strategy is divided into two steps. The first step involves the determination of the TI patterns; the second involves the projection of these patterns into the sample while performing EDOF imaging. A key benefit of this separation is that we can prioritize a different aspect of the performance of each step. In the first step we emphasize accuracy of TI pattern determination; in the second we emphasize imaging speed.

To characterize the performance of our strategy, we imaged a 300 μm thick GFP-labeled mouse brain slice. The EDOF range was set to approximately 150 μm, beyond which it becomes difficult to resolve neurons. We generated 60 different TI patterns throughout the EDOF range, corresponding to an average 2.5 μm axial range per pattern. This is roughly the same range as the conventional DOF of our microscope based on the NA of our objective, and thus roughly corresponds to the axial resolution of our system. The average fill factor of the TI patterns, defined by the ratio of “on” to total pixels, was about 0.02.

**Figure 3:**
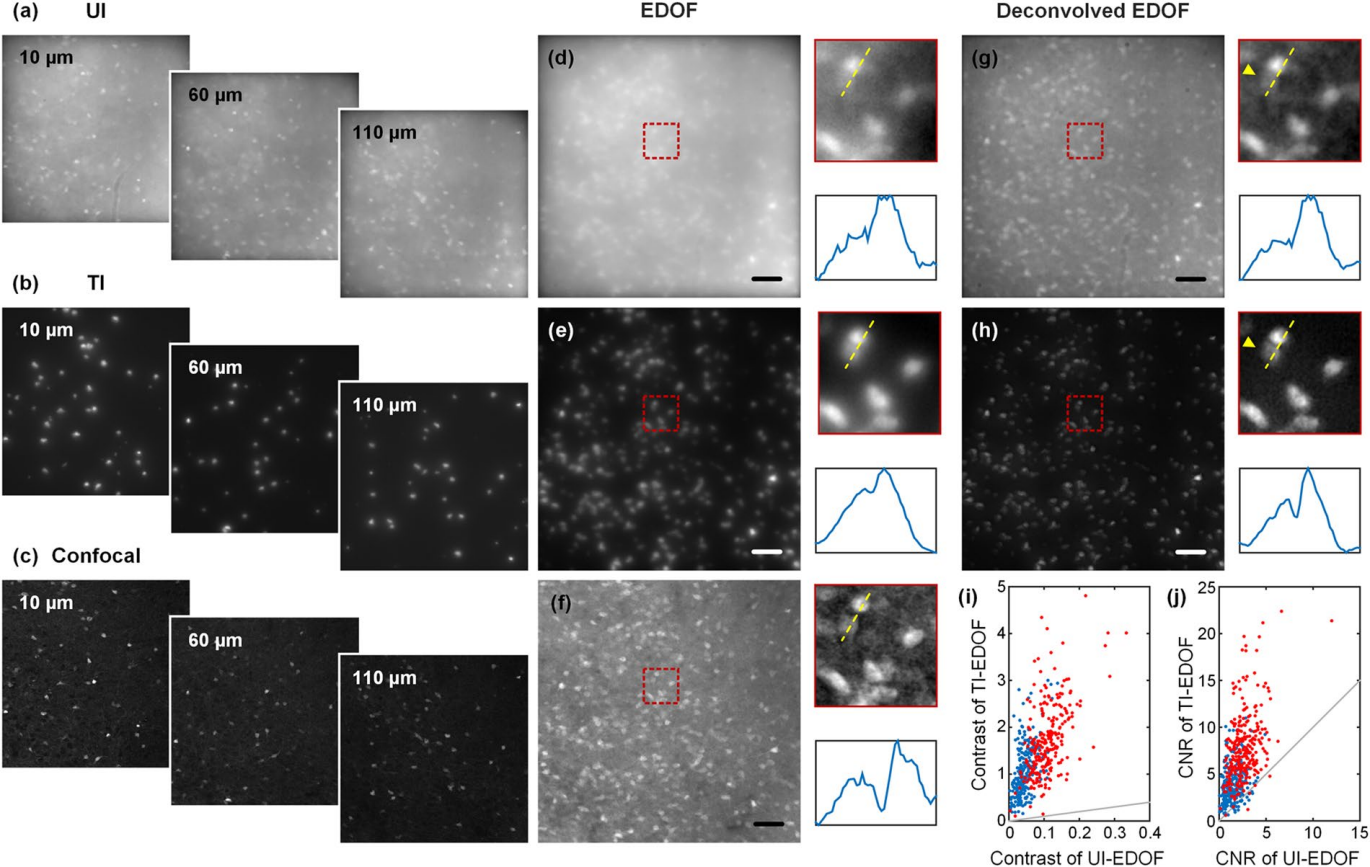
Images of a 300 μm thick brain slice at *z* = 10 μm, 60 μm and 110 μm acquired with UI (**a**), TI (**b**) and confocal microscopy (**c**). (**d**) and (**e**) are EDOF images spanning a 150 μm axial range acquired in a single shot with 40 ms exposure time with UI and TI respectively. (**f**) is the confocal EDOF image spanning the same 3D FOV. (**g**) and (**h**) are the deconvolved images of (**d**) and (**e**). The yellow arrow indicates a neuron that is almost indistinguishable from background in the deconvolved UI-EDOF image, but is clearly apparent in the deconvolved TI-EDOF image. (**i**,**j**) Contrast and CNR comparisons of UI- and TI-EDOF images, before (blue dots) and after (red dots) deconvolution. Gray lines indicate when the two cases have equal contrast/CNR. Scale bar, 50 μm.

We first compare the images acquired with UI and TI at discrete depths. Figure 3(a,b) shows images acquired at depths *z* = 10 μm, 60 μm and 110 μm. Those acquired with TI already exhibit a significant reduction in fluorescence background. The complete sequence of 60 patterns and their corresponding single layer UI and TI images are available in SI video 2. We then imaged the same volumetric region in a single shot over the continuous 150 μm axial range. The resulting EDOF images are shown in Fig. 3(d,e). We find that while background greatly increases in the UI-EDOF image, the TI-EDOF image continues to retain a relatively low background and high contrast.

To confirm that our system accurately captures sample features rather then producing artifacts arising from patterned illumination, we imaged the same volumetric region with a commercial confocal microscope (Olympus FV1000 with a Olympus UPlanSApo 10×/0.4 objective). Confocal images acquired at the same three depths in Fig. 3(a,b) are shown in Fig. 3(c). To compare EDOF images, we acquired a confocal *z*-stack over the same 3D FOV, comprising 75 images at 2 μm step intervals. We then summed these to synthesize a confocal EDOF image. The result is shown in Fig. 3(f). We observe that the TI-EDOF image, though more blurred than the confocal counterpart, retains most of the structural features of the neuronal cell bodies. Moreover, a large portion of featureless background present in confocal EDOF image has been removed with TI. In effect, this featureless background is suppressed because it is not illuminated.

We emphasize that EDOF intrinsically leads to image blurring. However, this blurring is well defined and thus reliably amenable to de-blurring with deconvolution, as described above. This is shown in Fig. 3(g,h), where we applied WH filtering to deconvolve both the UI- and TI-EDOF images. The UI-EDOF image was deconvolved with a WH filter whose depth parameter *D* = 150 μm. We observe that although the image contrast has been improved, there remains considerable background. Alternative deconvolution algorithms, such as nonlinear algorithms, might provide better results, but they are generally much more computationally intensive and difficult to implement in real time^35^. For the TI-EDOF image, we found that a WH filter with *D* = 60 μm yielded satisfactory results, as illustrated in Fig. 3(h). When compared with the confocal EDOF image, the deconvolved TI-EDOF image reveals similar details but with even less background. At present, we do not know how to best optimize the selection of *D* for TI-EDOF in an automated way, and we perform this optimization instead in a subjective manner by eye. We have found that a large range of *D* values seems to yield equally acceptable results, though these values are systematically larger than might be expected from the sparsity level of our TI. This may be due to the presence of tissue scattering which is not accounted for in our EOTF model (Eq. 2), which has a similar effect as the extension of *D* in EOTF, namely the attenuation high spatial frequencies thus causing image blurring. A comparison of how the choice of *D* affects the image quality is presented in Supplementary Information.

To quantify the improvement of image quality with our TI-EDOF method, we make use of two measures. The contrast of the images is defined by (*μ*_*s*_ − *μ*_*b*_)/*μ*_*b*_, where *μ*_*s*_ is the signal intensity (here averaged over neuron bodies) and *μ*_*b*_ is the background intensity (here obtained in the vicinity of the neurons). Similarly, the contrast-to-noise ratio (CNR), which is a reasonable measure of SNR in presence of strong background, is defined by (*μ*_*s*_ − *μ*_*b*_)/*σ*_*b*_, where *σ*_*b*_ is the standard deviation of background. Both contrast and CNR are calculated locally for each neuron. It should be noted that when the TI fill factor is small, *μ*_*b*_ is dominated by an offset in the camera readout. To correct for this, we systemically subtracted this offset from both *μ*_*s*_ and *μ*_*b*_.

We segmented out 254 neurons within the FOV, and compared their contrast and CNR with UI- and TI-EDOF imaging, both before (blue dots) and after (red dots) deconvolution–see Fig. 3(i,j). The average improvements of TI over UI are summarized in Table 1. Both TI and deconvolution separately lead to contrast and CNR enhancements. When combined, the enhancements become substantial.

**Table 1:** Average improvements on CNR and contrast.

	Contrast	CNR
UI-EDOF	1×	1×
Deconvolved UI-EDOF	2.91×	2.47×
TI-EDOF	28.99×	4.19×
Deconvolved TI-EDOF	48.96×	8.82×

**Figure 4:**
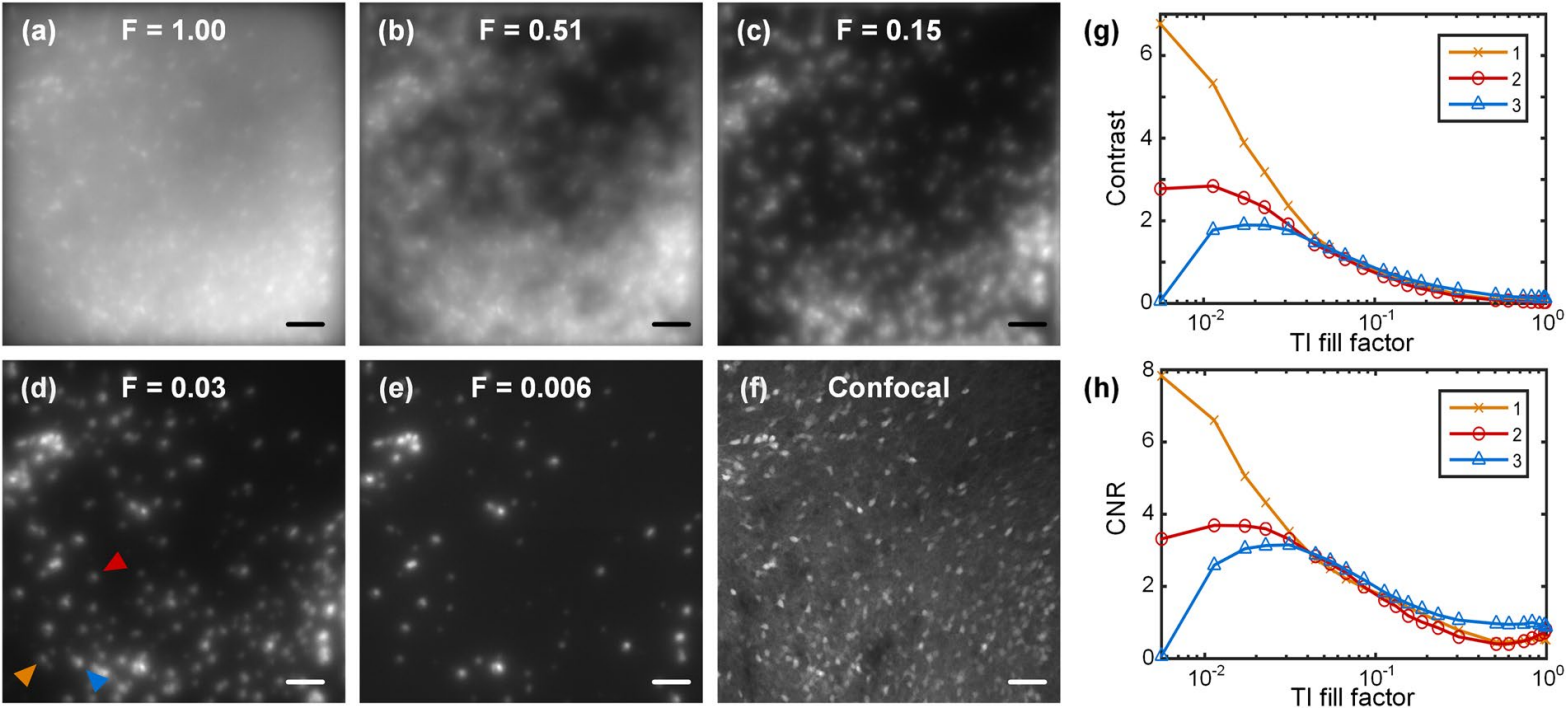
Influence of TI fill factor F on TI-EDOF images. From (**a**–**e**), TI-EDOF images when TI fill factor is 1.00, 0.51, 0.15, 0.03, 0.006 respectively. (**f**) is the confocal EDOF image spanning the same 3D FOV. (**g**) Contrast and (**h**) CNR of three cells as a function of TI fill factor. Cells marked in (**d**) with colored arrows are represented in (**g**,**h**) with the same colored lines. Markers on plots indicate measured data points. Scale bars in (**a**–**f**) are 50 μm.

We then investigated the effect of TI sparsity (fill factor) on image quality. We imaged another 300 μm brain slice with an EDOF range of 150 μm. By changing the threshold level *T* used to generate the TI patterns (see Supplementary Information), we captured TI-EDOF images with varying TI fill factors ranging from 0.006 to 1. Some examples are shown in Fig. 4(a–e). As a reference, Fig. 4 shows a confocal EDOF image acquired over the same 3D FOV (obtained from a stack of 60 confocal images over a 150 μm axial range at 2.5 μm intervals, with an Olympus UPlanSApo 20×/0.75 objective). We observe that low TI sparsity (high fill factor) produces significant out-of-focus background, whereas high TI sparsity (low fill factor) fails to capture many of the neurons within the FOV. In practice, an optimal comprise seems to be a fill factor commensurate with that of the neurons themselves. In this example, a TI fill factor around 0.03 yielded the best results, as illustrated in Fig. 4(d).

In this same example, we quantitatively measured the local contrast and CNR of three representative neurons indicated in Fig. 4(d). The dependences of these on TI fill factor are plotted in Fig. 4(g,h). For neuron # 1, the contrast and CNR continued to increase as we decreased the TI fill factor, owing to the reduction in background and associated shot noise. But for neurons # 2 and # 3, the contrast and CNR eventually decrease when the TI fill factor becomes too low, which can be attributed to the clipping of signal when the TI pattern is too sparse. In general, the contrast and CNR of neurons increases with decreasing TI fill factor provided the neurons remain fully comprised within the TI patterns.

A heuristic calculation of the improvements in contrast and CNR is presented in Supplementary Information.

### Dynamic imaging

Ultimately, the main advantage of our TI-EDOF technique is the ability to achieve high-contrast volumetric imaging at high speeds. Once the calibration procedure is completed and the TI patterns are loaded into computer memory, the streaming of these patterns for TI-EDOF imaging can occur at the highest frame rate allowed by our DMD. An important application where speed is a concern is functional imaging, specifically neuronal imaging in the mouse brain.

To demonstrate the benefits our TI-EDOF in functional imaging, we performed *in vivo* imaging of the calcium dynamics in the striatum area of a mouse brain. The mouse was fitted with a custom imaging window, and injected with AAV9-Syn-GCaMP6f (see Methods for details). During imaging, the mouse was awake, head-fixed, constrained and mounted on an articulated-base ball stage.

To compare *in vivo* calcium signals obtained with UI and TI, we modified our control software to perform interleaved acquisitions. The details of our synchronization are described in Supplementary Information. The final imaging was performed at 10 Hz frame rate with 40 ms effective exposure time for both types of illuminations.

The total EDOF range was set to approximately 200 μm. It should be noted that we did not always penetrate 200 μm into the brain, since the surface of the brain was somewhat tilted within the FOV, which is a common occurrence when imaging mice brains. To minimize motion artifacts, we limited ourselves to 20 different TI patterns to span the full EDOF range, with an average fill factor of 0.16. A complete sequence of these 20 pattern is presented in SI video 3.

**Figure 5:**
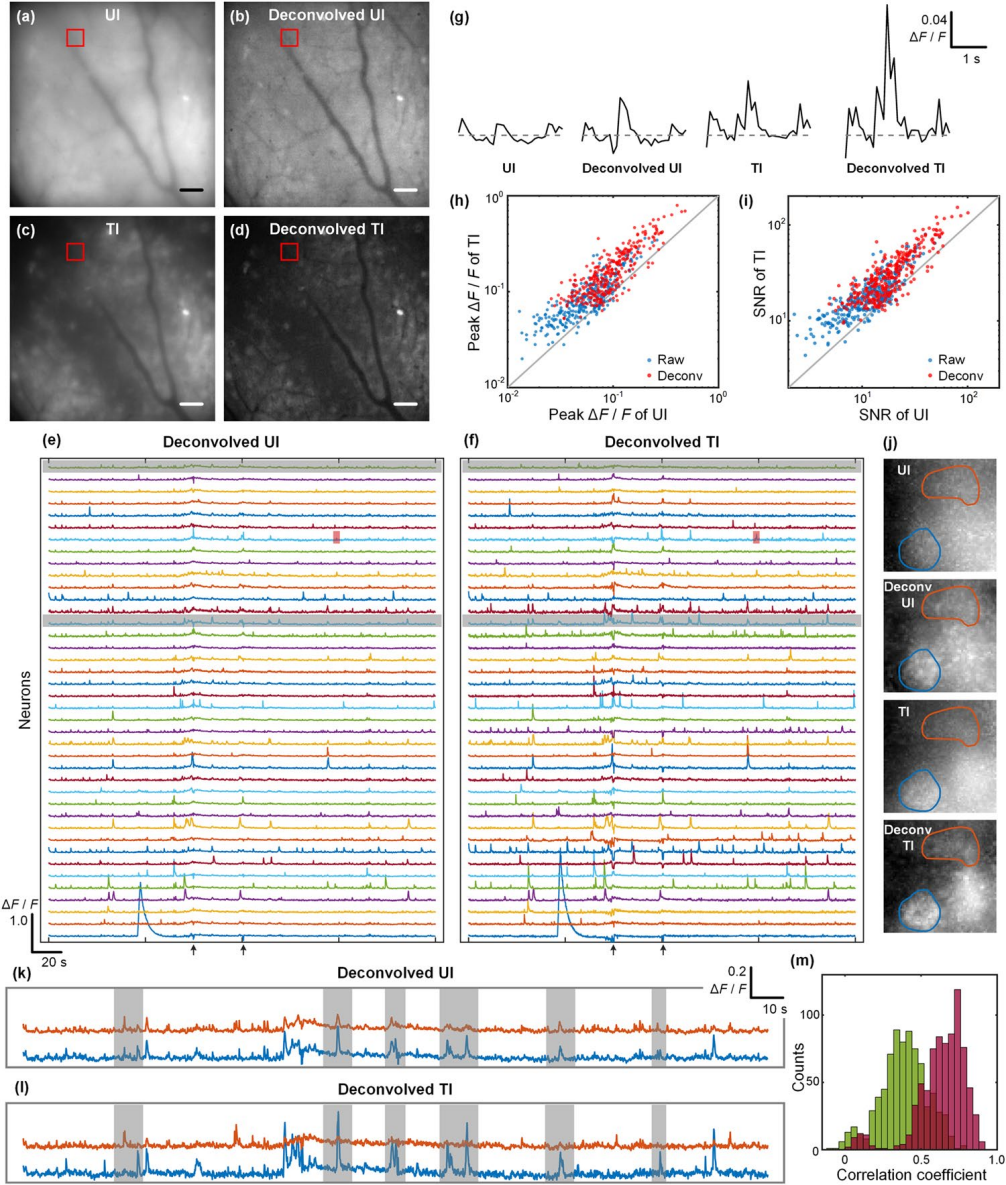
One frame from the videos acquired with UI (**a**) and TI (**c**). (**b**) and (**d**) are the deconvolved images of (**a**) and (**c**), respectively. The imaging volume is approximately 500 × 500 × 200 μm. (**e**,**f**) Calcium traces of 40 neurons obtained from the deconvolved UI and TI videos. Arrows indicate times when motion artifacts occur (see Supplementary Information Fig. S8). (**g**) Expanded view of the calcium transients from the red shaded region in (**e**,**f**). Gray dashed line indicates Δ*F*/*F* = 0. (**h**,**i**) Comparison of Δ*F*/*F* peak signal (**h**) and SNR (**i**) acquired with UI and TI, without (blue dots) and with (red dots) deconvolution. (**j**) Maximum projection image of two neighboring neurons from the rectangular box region in (**a**–**d**). From top to bottom: UI, deconvolved UI, TI, deconvolved TI. The calcium traces of these two neurons are shown in (**k**,**l**), which correspond to the gray shaded calcium traces in (**e**,**f**). Gray shaded region in (**k**,**l**) illustrate when signal crosstalk present in deconvolved UI is reduced or eliminated with deconvolved TI. (**m**) Histogram of correlation coefficient between pairs of neurons in deconvolved UI-EDOF video (red) and deconvolved TI video (green). Scale bars in (**a**–**d**) are 50 μm. (The corresponding calcium traces in (**e**,**f**,**k**,**l**) and histogram of correlation coefficient in (**m**) before frame-by-frame deconvolution are shown in Supplementary Information).

We recorded two 4 minute videos by interleaving UI and TI. Both videos were deconvolved on a frame-by-frame basis according to Eq. 3. The UI video was deconvolved with *D* = 200 μm and the TI video with *D* = 150 μm. Figure 5(a,c) shows one frame from the raw videos and Fig. 5(b,d) shows these frames after deconvolution. In both cases, TI-EDOF yields higher contrast and more structural information. The complete videos for all cases are presented in SI video 4.

We isolated 40 active neurons within the FOV, and plotted their relative signal levels Δ*F*/*F* (Δ*F* = *F*_*signal*_ − *F*_*baseline*_; *F* = *F*_*baseline*_) over the recorded period. The results for both videos before and after deconvolution are presented in Figs 5(e,f) and S7. We observe that while the signals from UI and TI are correlated, TI typically provides higher signal for the same calcium transients, facilitating the identification of weak calcium transients. Figure 5(g) shows an example where a calcium transients is nearly invisible with UI, but becomes readily apparent in deconvolved TI.

We then analyzed 324 calcium transients obtained during the recording. Figure 5(h) compares scatter plots of the peak Δ*F*/*F*’s obtained with UI and TI, both before (blue dots) and after deconvolution (red dots). On average, TI yielded an average increase in peak signal over UI for about 58% before and 81% after deconvolution. And deconvolution yielded an average increase in peak signal of about 74% for UI and 100% for TI. To characterize the sensitivity of our measurements, we define the SNR associated with the calcium traces as the peak Δ*F*/*F* divided by the standard deviation during resting activity. The SNR for the 324 calcium transients is shown in Fig. 5(i). Compared to UI, TI enhances SNR on average by 65% before deconvolution and 76% after deconvolution. Deconvolution itself enhances SNR on average by 68% in the case of UI and 79% in the case of TI.

By definition, an increase in SNR implies an increase in information content. This is manifested in TI-EDOF in several ways. One example is in the reduction of spurious crosstalk between neuronal signals. Specifically, out-of-focus blur can cause the signals from neighboring neurons to overlap, leading to ambiguities in signal identification. Such spurious crosstalk is exacerbated with longer EDOF depths, where blurring becomes more significant. Clear examples of such crosstalk are apparent in Fig. 5(k), showing the signals from two nearby neurons acquired with UI. In contrast, because TI suppresses out-of-focus fluorescence, the crosstalk (Fig. 5(l)) is significantly reduced. To estimate the reduction in spurious crosstalk, we analyzed the normalized cross-correlation of calcium traces between all pairs of neurons in the UI and TI videos. Figure 5(m) shows the distributions of these correlation coefficients for post-deconvolution videos. While it is expected that different neurons should be correlated to some extent, TI led to a marked reduction in cross-correlation compared to UI. Specifically, the average cross-correlation decreased from 0.82 before deconvolution and 0.64 after deconvolution in the UI video, to 0.66 before deconvolution and 0.38 after deconvolution in the TI video. These results suggests that much of the cross-correlation observed in the UI video was not physiological in origin, but rather was the result of spurious blur-induced crosstalk.

Another example of how TI leads to improved information content is in neuron identification. When imaging task-performing animals over large FOVs for long periods of time, one can easily monitor hundreds or even thousands of active neurons. Automated segmentation and signal extraction algorithms are thus required to distinguish these neurons. Such algorithms can perform poorly with UI videos, but benefit from the higher contrast and lower crosstalk of TI videos. For example, the method of independent component analysis relies on the signal independence between different neurons. Such independence is undermined by blur-induced crosstalk. Similarly, the method of constrained non-negative matrix factorization (CNMF)^36^, suffers when image contrast is low. By alleviating spurious temporal cross-correlations and enhancing spatial image contrast, TI reduces the ambiguities that arise when separating neighboring cells or cells from background in the initialization and iterative merging/discarding steps, resulting in more reliable reconstructions. An example where the CNMF successfully identifies two neighboring neurons with TI but fails with UI is presented in Supplementary Information.

**Figure 6:**
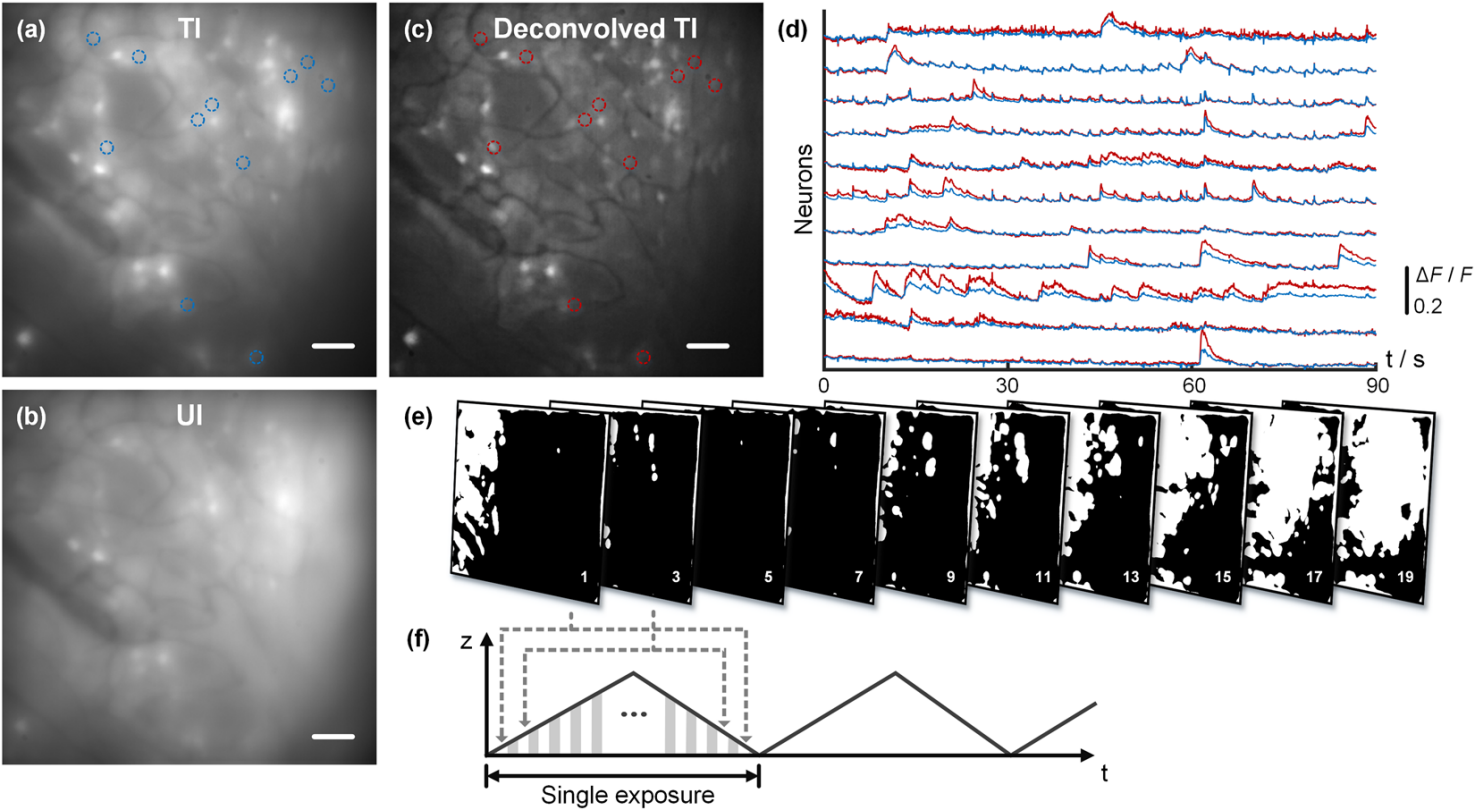
One frame from a video acquired with (**a**) TI-EDOF and (**b**) UI-EDOF imaging across 500 × 500 × 100 μm FOV. (**c**) Deconvolved frame of (**a**). (**d**) Calcium traces of from the raw video (blue line) and frame-by-frame decon-volved video (red line). The positions of the associated neurons are indicated in (**a**) and (**b**). (**e**) 10 out of 20 TI patterns used for imaging. (**f**) Synchronization scheme. Scale bars in (**a**–**c**) are 50 μm.

Finally, we demonstrate the speed capacity of our system by performing video-rate volumetric functional imaging. Specifically, we imaged another region of the same mouse brain using a 3D FOV about 500 × 500 × 100 μm at 30 Hz frame rate for 90 s. We generated 20 TI patterns with 0.30 average fill factor, some of which are shown in Fig. 6(e). The complete 20 patterns are presented in SI video 5. The ETL was driven by a triangular wave, and each TI pattern was displayed twice at the same depth during every exposure, as described in Fig. 6(f).

A sample frame from the recorded video is shown before (Fig. 6(a)) and after (Fig. 6(c)) deconvolution using *D* = 60 μm. For comparison, Fig. 6(b) shows the same 3D FOV acquired with UI-EDOF, which manifestly contains much higher background. We found that 11 neurons were active during the recording, and plotted their relative calcium signals in Fig. 6(d), where blue represents the signal from the raw video and red represents the signal after frame-by-frame deconvolution. The recorded videos both before and after deconvolution are presented in SI video 6.

## Discussion

In summary, we have developed a technique for high speed EDOF imaging with improved contrast and SNR. With our current setup, the maximum achievable 3D FOV is ~500 × 500 × 240 μm. This FOV could be made larger with the use of lower magnification objectives. Alternatively, our imaging resolution could be increased with the use of a higher NA objective. While this would lead to more rapid out-of-focus blurring, which is generally deleterious to EDOF imaging, such blurring is largely suppressed by TI-EDOF. Our maximum frame rate is currently 100 Hz, limited by the ETL scan rate, though this rate could be increased by making use of alternative focal scan mechanisms. For example, we previously demonstrated the potential for kilohertz EDOF imaging when using a deformable mirror^32^.

Compared to imaging methods that make use of structured illumination, we offer significantly improved speed, since, once the TI patterns are loaded into memory, only a single TI pattern is delivered per focal depth rather than a sequence. Moreover, we offer improved contrast and SNR. The reason for this is that, to perform fast imaging, SIM requires dense illumination structures to fill the FOV with as few structures as possible (a minimum of three). In contrast, the calibration step in our technique can make use of sparse SIM illumination structures, since speed is generally not an issue during this step. Sparse illumination in SIM leads to improved structure visibility in dense samples, and hence improved contrast, SNR, and utilization of the camera dynamic range for the identification of in-focus sample features. Compared also to methods that make use of spatially controlled illumination^37–39^, our technique offers similar advantages of reduced light dosage, presumably leading to reduced photobleaching and phototoxicity. In the case where photobleaching or phototocicity scale with illumination, we expect this reduction to be proportional to the fill factor of our TI patterns.

But our system is not without drawbacks. Its main drawback is a susceptibility to motion artifacts. If a neuron shifts in and out of its dedicated TI area, for example because of a sudden tissue twitch, it will produce signal variations that are not related to neuronal activity (see times indicated by arrows in Fig. 5(e,f)). But such motion artifacts are easy to identify, since they manifest themselves as sudden decreases in neuronal signal that occur simultaneously throughout the FOV. This problem can be alleviated by using TI patterns that provide extra wiggle room to allow for sample motion^6^, though at the expense of pattern sparsity (in any case, we systematically provide some degree of wiggle room–see Supplementary Information). Alternatively, active tracking of the sample can allow the TI patterns to be updated when necessary^40^.

Another drawback of our system comes from the use of a threshold in the calibration step to discriminate in-focus from out-of-focus sample features. The greater this threshold, the more sparse our TI patterns, but the greater our chances of failing to capture weakly fluorescent sample features. Inevitably, the use of a threshold means that some weak features in the sample will be overlooked (see Fig. 4). Also, with our current implementation, neurons may not be detected if they are inactive during calibration and their fluorescence is too weak. This problem can be resolved with dual-color labeling that combines GCaMP with another static reporter at a different wavelength for cell identification.

We note finally that, DMD-based illumination has been widely adopted in optogenetic applications to deliver light to targeted neurons^39–41^. These applications have been limited mostly to 2D planes. Here, by using a fast axial scanning mechanism, our system can deliver light at targeted locations within a 3D volume. In principle, we can also restrict our 3D TI pattern to only specific neurons of interest. Thus our system can potentially be useful for high spatio-temporal resolution extended volume optogenetic applications.

But the key advantage of our technique which we have demonstrated here is speed, and the ability to perform high resolution and high contrast imaging over extended volumes at video rate. We anticipate that this advantage will be useful for the study of large-scale neuronal circuitry.

## Methods

### Mouse Preparation

All animal procedures were approved by the Boston University Institutional Animal Care and Use Committee, and the experiments were carried out in accordance with the approved guidelines. Female C57BL/6 mice (Taconic, Hudson, NY), 8–12 weeks old, were first implanted with a custom imaging window, targeting striatum area (AP: +0.5, ML: 1.8, DV: −1.6). The custom imaging window was built with a stainless steel imaging cannula (OD: 3.17 mm, ID: 2.36 mm, height: 2 mm; Small Parts: B004TUE45E), a circular coverslip (size 0; OD: 3 mm, Warner Instruments: CS-3R-0), and an infusion guide cannula (26 gauge, C135GS4, Plastics, Roanoke, VA). The coverslip was fit onto the imaging cannula by using a UV-curable optical adhesive (Norland Products), and the infusion guide cannula was attached to the imaging cannula at 45 degree angle with its tip next to the coverslip. During the surgery, a custom aluminum head-plate was also attached to the skull. After recovery, animals were injected with 500 nl AAV9-Syn-GCaMP6f.WPRE.SV40 (titer: 6.6 × 1012 GC/ml, University of Pennsylvania Vector Core) with a 10 μl syringe (701 N; Hamilton Company, Reno, NV) and a 33 gauge needle (C135IS4; Plastics, Roanoke, VA). The injection was controlled by a microinfusion pump (UltraMicroPump3-4; World Precision Instruments, Sarasota, FL) at the rate of 100 nl/min.

## Electronic supplementary material

Supplementary Information

SI video 1

SI video 2

SI video 3

SI video 5

SI video 4

SI video 6
