# Supplementary material for: Video-rate volumetric neuronal imaging using 3D targeted illumination
Source: Sci Rep. 2018 May 21;8:7921. doi: 10.1038/s41598-018-26240-8 (PMC5962542; doi:10.1038/s41598-018-26240-8)
Supplement: Supplementary file 1 — Supplementary Information [file 41598_2018_26240_MOESM1_ESM.pdf]

Video-rate volumetric neuronal imaging using 3D targeted illumination: supplementary information

Sheng Xiao[1,*], Hua-an Tseng[2], Howard Gritton[2], Xue Han[2], and Jerome Mertz[2]

[1]Department of Electrical & Computer Engineering, Boston University, 8 Saint Mary's St., Boston, Massachusetts 02215, USA
[2]Department of Biomedical Engineering, Boston University, 44 Cummington Mall, Boston, Massachusetts 02215, USA
[*]Corresponding author: shengx@bu.edu

ABSTRACT

This document provides supplementary information to "Video-rate volumetric neuronal imaging using 3D targeted illumination".

1 TI pattern generation

The SIM procedure for generating TI patterns at every depth z is presented in Algorithm 1 and also illustrated in Fig. 1. At every depth z, we project a series of checkerboard-like illumination patterns with a predefined feature size s and pitch p, both of which can be increased for thick, scattering samples to ensure that the in-focus sample features in the structured illumination images are resolvable. A total number of $N = (p/s)^2$ illumination structures are required to cover the complete FOV. For each illumination structure, we obtain a raw image $I^n_{raw}(\vec{\rho},z)$, where $\vec{\rho}$ is represent lateral coordinates, and $n = 1,2,...,N$ is the structure index. As is the principle of SIM, in-focus features vary with high contrast while out-of-focus features vary with low contrast. A variety of algorithms can be used at this point to extract in-focus from out-of-focus sample features. In our case, we estimated the image background $I^n_{lp}(\vec{\rho},z)$ by applying a Gaussian low pass filters $LP(\vec{\rho})$ to each raw image, such that $\int LP(\vec{\rho})d^2\vec{\rho} = 1$. The complementary high-passed images $I^n_{hp}(\vec{\rho},z)$ were also calculated. We then inferred the presence of in-focus sample features by estimating the envelope image $I^n_{env}(\vec{\rho},z)$ associated with each $I^n_{hp}(\vec{\rho},z)$. Assuming our background noise is dominated by shot noise, the corresponding SNR of these envelope images scales as $SNR^n(\vec{\rho},z) = I^n_{env}(\vec{\rho},z)/\sqrt{I^n_{lp}(\vec{\rho},z)}$. We then applied a threshold T on $SNR^n(\vec{\rho},z)$ to create a binary pattern $M^n(\vec{\rho},z)$ for the nth structured illumination image. An advantage of thresholding on $SNR^n(\vec{\rho},z)$ instead of directly on $I^n_{env}(\vec{\rho},z)$ is that the threshold value T depends solely on SNR, and we can thus adjust illumination intensity and exposure time without altering T. The final TI pattern $M(\vec{\rho},z)$ at depth z is obtained by performing a pixelwise OR for all $M^n(\vec{\rho},z), n = 1,2,...,N$.

Algorithm 1 TI pattern generation at depth z

Input: Structured illumination images $I^n_{raw}(\vec{\rho},z)$, $n = 1,2,...,N$
for n = 1, 2, ..., N **do**
  $I^n_{lp}(\vec{\rho},z) = LP(\vec{\rho}) \otimes I^n_{raw}(\vec{\rho},z)$ ▷ Low pass
  $I^n_{hp}(\vec{\rho},z) = I^n_{raw}(\vec{\rho},z) - I^n_{lp}(\vec{\rho},z)$ ▷ High pass
  $I^n_{env}(\vec{\rho},z) = \sqrt{LP(\vec{\rho}) \otimes [I^n_{hp}(\vec{\rho},z)]^2}$ ▷ Envelope extraction
  $SNR^n(\vec{\rho},z) = I^n_{env}(\vec{\rho},z)/\sqrt{I^n_{lp}(\vec{\rho},z)}$
  $M^n(\vec{\rho},z) = SNR^n(\vec{\rho},z) > T$ ▷ Thresholding
end for
$M(\vec{\rho},z) = M^1(\vec{\rho},z) \vee M^2(\vec{\rho},z) \vee \cdots \vee M^N(\vec{\rho},z)$
return $M(\vec{\rho},z)$

By varying the threshold T we can control the TI pattern sparsity, as shown in Fig. 2. The sample and TI patterns are the same as in Fig. 1. In general, low sparsity TI patterns unnecessarily capture background, which undermines the final image contrast and SNR. As sparsity increases, the TI patterns are better confined to only in-focus neurons, thus helping to reduce

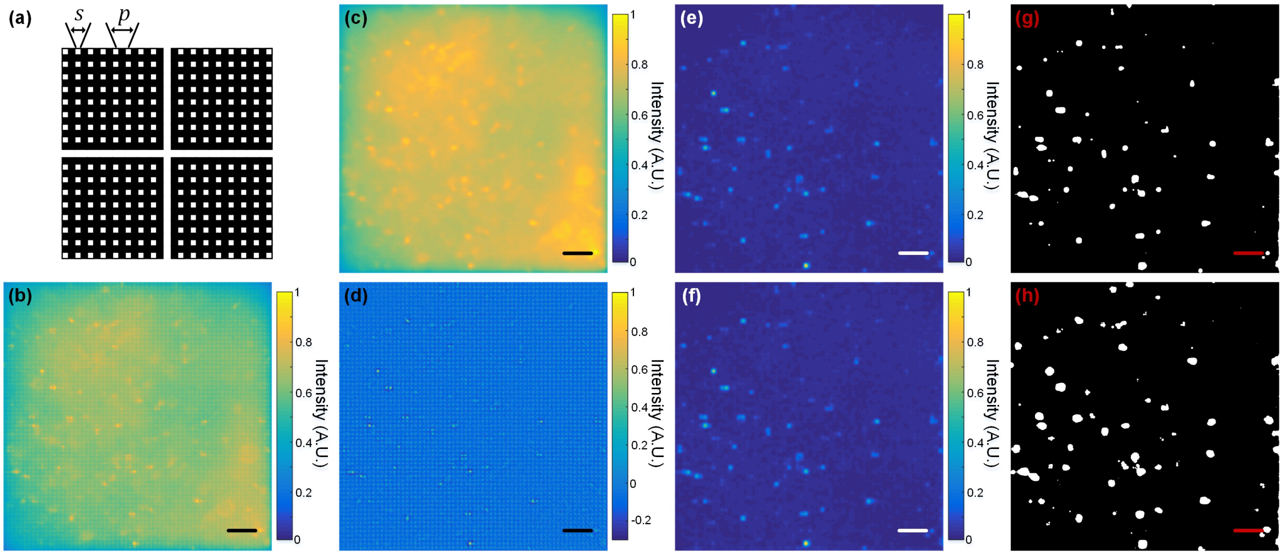

Figure 1. (a). Illustration of sparse SIM illumination structures. These four structures cover the complete lateral FOV. (b). Structured illumination image $I^1_{raw}(\vec{\rho}, z_0)$ of a thick brain slice at depth z_0. (c). Low-passed image $I^1_{lp}(\vec{\rho}, z_0)$. (c). High-passed image $I^1_{hp}(\vec{\rho}, z_0)$. (e). Envelope-associated image $I^1_{env}(\vec{\rho}, z_0)$. (f). SNR image $SNR^1(\vec{\rho}, z_0)$. (g). TI pattern $M^1(\vec{\rho}, z_0)$ generated from $I^1_{raw}(\vec{\rho}, z_0)$. (h). Final TI pattern $M(\vec{\rho}, z_0)$ by performing pixelwise OR on all $M^n(\vec{\rho}, z_0), n = 1, 2, 3, 4$. All scales bars are 50 μm. A.U.: arbitrary unit.

background while still providing some wiggle room about the neurons to allow for small sample motion. As sparsity further increases, the TI patterns become more tightly confined, and we attain maximal SNR when the TI patterns exactly encompass each neuron, though at the cost of being sensitive to motion artifacts. Any further increase in sparsity encroaches on the in-focus fluorescence, causing reduction in contrast and SNR.

The speed for SIM acquisition will be limited by the maximum camera frame rate, but our typical imaging speed is 30 Hz. The total acquisition time will depend on the number of SIM images required, which depends on the sparsity of the SIM pattern and the total number of TI patterns used.

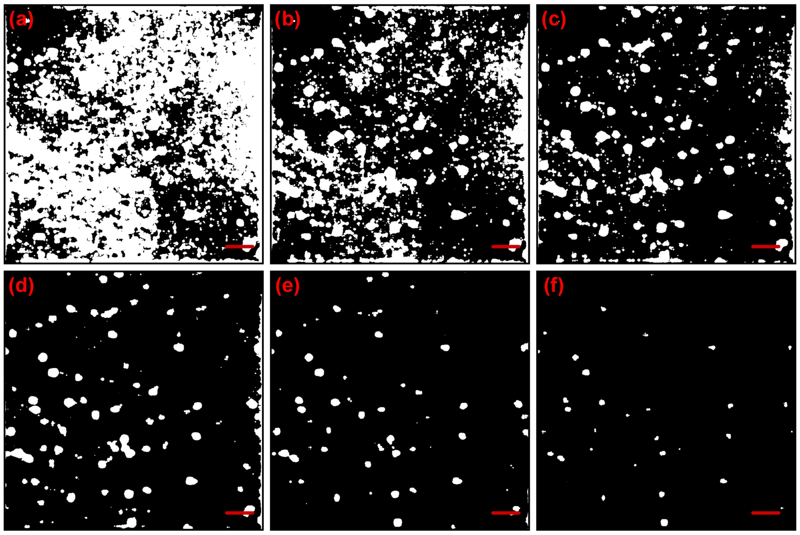

Figure 2. TI patterns with varying fill factor. From (a) to (f) the TI fill factors are 0.62, 0.28, 0.13, 0.066, 0.026, and 0.0077. Scale bars are 50 μm.

2 Deconvolution

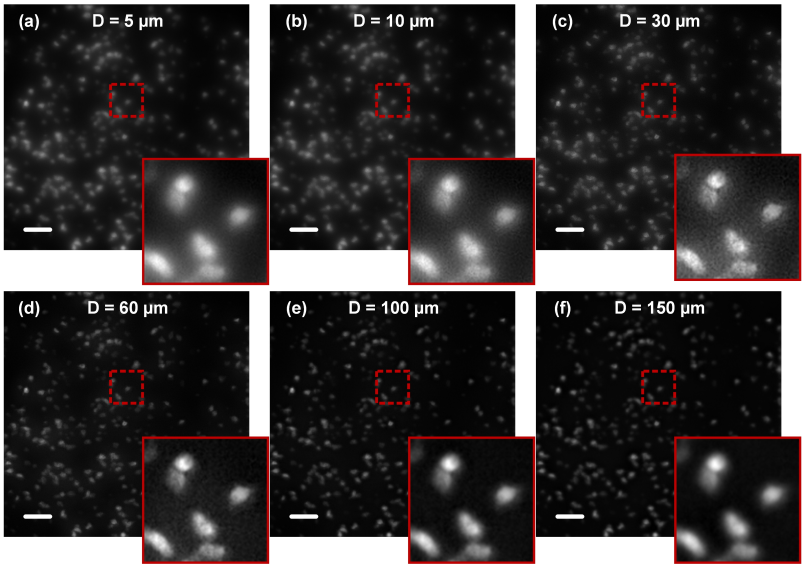

Figure 3. Deconvolved images of Fig. 3(e) when applying different D. From (a) to (f), the depth parameter was set to 5, 10, 30, 60, 100, and 150 µm. Scale bars are 50 µm.

Here we give a few examples on how the deconvolution parameter D affects the deconvolved images. By deconvolving the image in Fig. 3(e) with the depth parameter D = 5, 10, 30, 60, 100, 150 µm, we obtain the resulting images in Fig. 3. It can be seen that the images still contain too much background with a small D. And for large D's, while background has been suppressed, some image details are also lost. For this example, D around 60 µm yields the best results. However, we believe that D needed to be increased for samples that are more scattering, such as when we are doing *in vivo* brain imaging.

The presence of regularization parameter is used to suppress high frequency noise. Generally, higher regularization needs to be applied for images with lower SNR, without which the deconvolved image tends to noisy. But too much regularization will affect the accuracy of the PSF estimation, and also might suppress high frequency sample features. So in practice, the regularization parameter is usually set to be $\varepsilon \ll 1$.

3 Nonnegative Matrix Factorization

We tested CNMF algorithm on both UI and TI videos to verify the performance improvement obtained with TI. The data is a small cropped region from the 4 minute videos obtained with interleaved UI and TI acquisition. Within this region, there are two overlapping neurons. (Note that frame 881 to frame 910 have been removed from the video due to obvious motion artifacts.)

We first manually segmented the neurons in the UI and TI videos (Fig. 4(a, b)), and plotted their calcium response in Fig. 4(e, f). We then applied the CNMF algorithm to the videos. The autoregression order was set to 0. While the CNMF algorithm worked flawlessly with our deconvolved TI video (Fig. 4(c, g)), we encountered several problems when applying it to the deconvolved UI video. Firstly, the greedy initialization worked poorly. Only one of the neurons had its location correctly identified, and we needed to manually set the location of the other one. Secondly, even after manually initializing the components to their correct locations, the dimmer neuron tended to be merged into the background or to the neighboring stronger neuron. We managed to prevent this merging process by increasing the correlation-based merging threshold. Thirdly, the dimmer neuron found by CNMF was very noisy due to the low contrast, as shown in Fig. 4(d). Because too little pixel weight was assigned this neuron, its calcium spike is almost invisible (orange trace in Fig. 4(h)).

4 Registration

When generating TI patterns, the camera and the DMD pixels must be registered to correct for system misalignment and pixel size mismatch. We first project a checkerboard pattern onto a thin planar fluorescent sample and capture an in-focus image. We then match the checkerboard coordinates between the projected and detected images, and a projective transformation is estimated using the MATLAB *fitgeotrans* function. This transformation is applied to all detected images in the main manuscript.

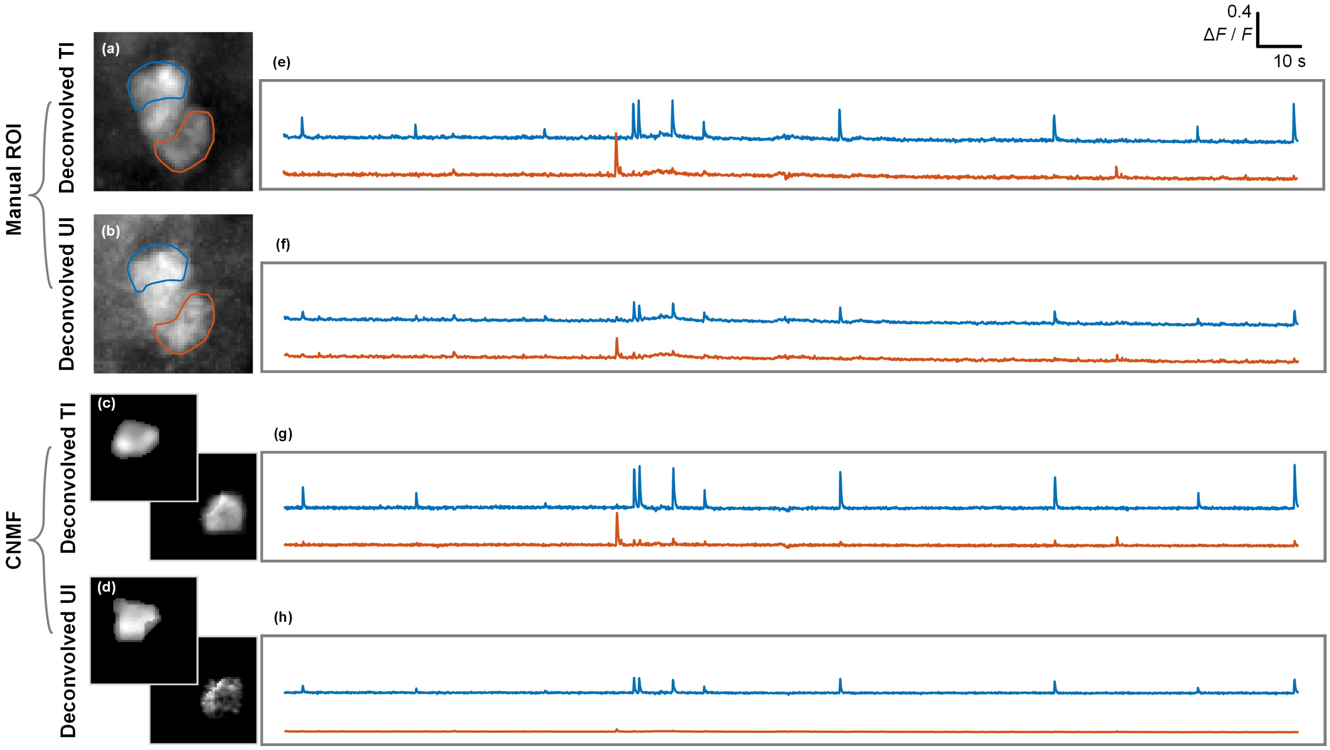

Figure 4. (a,b) Maximum projection images of deconvolved UI and TI video. Blue and orange curve represent manual segmentation of these 2 neurons. Their corresponding calcium traces are shown in (e,f). (c,d) shows the spatial footprints of 2 neurons found by CNMF algorithm on deconvolved UI and TI videos. Their corresponding temporal footprints are present in (g) and (h) respectively.

5 Device Synchronization

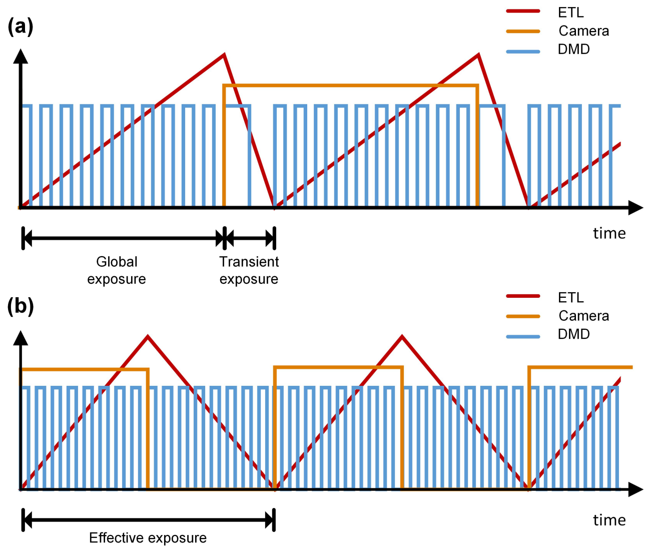

Figure 5. Synchronization of ETL, camera and DMD. (a). Synchronization scheme for interleaved acquisition. (b). Synchronization scheme for video-rate acquisition. The camera operates in overlapping exposure mode. The rising edge on the camera trigger pulse represents the start of the next exposure. The rising edge on the DMD trigger pulse represents a frame transition on the DMD.

To deliver the correct TI pattern to its targeted depth, all devices must be properly synchronized. In the manuscript, we use two different synchronization schemes, as illustrated in Fig. 5. For the interleaved acquisition, to avoid rolling shutter artifacts we only project TI patterns during the camera global exposure time. During the transient exposure time when consecutive frames overlap, all the DMD pixels are set to ”off”, which effectively blanks the illumination. In this case, we scan the ETL with a 20 Hz sawtooth wave whose rising slope spans the 40 ms global exposure and falling slope spans the 10 ms transient exposure.

For video-rate imaging, because the TI pattern sequence, camera and ETL operate at the same frequency, we do not need to be concerned about rolling shutter effect from the camera. In this case, the ETL was controlled with a 30 Hz triangular wave. The camera exposure time starts at the beginning of each triangular wave. Each TI pattern was displayed twice, symmetrically about the crest of the triangular drive signal upon every EDOF exposure.

In both cases, the DMD projects every TI pattern an equal amount of time. Because of the slight nonlinear response of the ETL, each pattern may cover slightly different axial scan distances. A temporal advance is added to the ETL control signal in order to compensate the ETL response latency. A custom MATLAB program controls device synchronization and image acquisition with a National Instruments DAQ card (NI PCI-6251).

6 Theory

We provide a heuristic estimate of the improvements in contrast (equated with signal-to-background ratio (SBR)) and SNR when performing TI-EDOF. We assume that the sample has thickness L and that the fluorescent sample features of interest are distributed throughout the sample with an average fill factor d_o. We also assume the TI patterns have a fill factor d_i.

Consider signals from a single layer of thickness $\delta z = \lambda/NA^2$ defined by the conventional microscope depth of field (λ is the wavelength and NA is the objective numerical aperture). When the associated TI pattern is focused onto this layer, the illumination is preferentially directed toward in-focus sample features. The fluorescence signal is then proportional to the overlap area between the illumination pattern and the in-focus features:

$$F_s = A\delta z \min(d_o, d_i) \tag{1}$$

where A is a constant taking in account lateral FOV, stain level, fluorescence quantum yield, etc. The fluorescence background produced by all other out-of-focus layers within the sample volume, assuming out-of-focus illumination becomes uniform due to blurring, is given by

$$B_1 = Ad_od_i(L - \delta z). \tag{2}$$

Because we perform a focal sweep within a single camera exposure, when the focus is scanned away from the signal layer, all collected fluorescence constitutes additional background. If the EDOF scan range D satisfies the relation $\delta z \ll D \leq L$, this additional background can be estimated to be

$$B_2 = Ad_od_iL\left(\frac{D - \delta z}{\delta z}\right). \tag{3}$$

We can estimate the final SNR by $\mathrm{SBR} = F_s/(B_1 + B_2)$. Assuming the system is shot-noise limited, the final signal-to-noise ratio scales as $\mathrm{SNR} = F_s/\sqrt{F_s + B_1 + B_2}$. From the above equations, these become:

$$\mathrm{SBR} = \frac{\delta z^2}{LD} \cdot \frac{\min(d_o, d_i)}{d_od_i} \tag{4}$$

$$\mathrm{SNR} = \sqrt{A\delta z}\frac{\delta z}{\sqrt{LD}} \cdot \frac{\min(d_o, d_i)}{\sqrt{d_od_i}} \tag{5}$$

Fig. 6 shows how SBR and SNR vary with TI fill factor. The general trends roughly corroborate our results in Fig. 4(g,h). As the TI fill factor decreases, the background and its associated shot noise decrease because of the decrease in illumination impinging on out-of-focus features. The in-focus signal, on the other hand, remains unchanged provided $d_i > d_o$, in which case the TI patterns fully comprise the in-focus features. Both SBR and SNR thus increase. However, when $d_i < d_o$, because the TI patterns no longer fully comprise the in-focus feature, the in-focus signal becomes clipped. In this case, though the background still decreases, SBR remains unchanged and SNR begins to decrease. The optimal TI fill factor is given by $d_i = d_o$, when the TI patterns exactly coincide with the in-focus sample features.

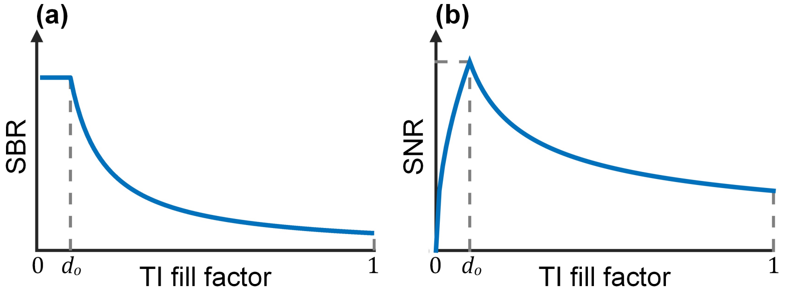

Figure 6. General trends of SBR (a) and SNR (b) as a function of TI pattern fill factor.

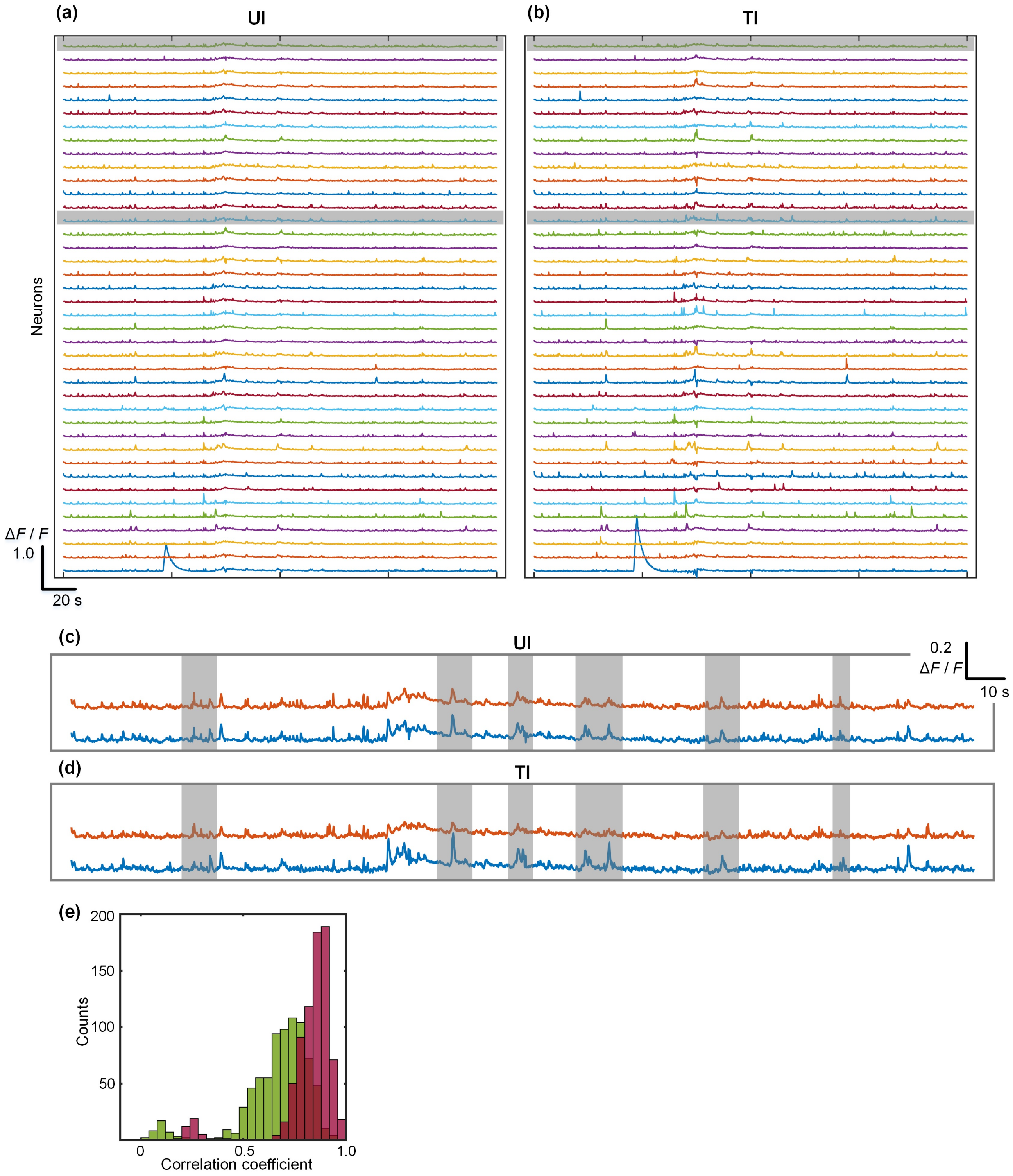

Figure 7. (a,b) Calcium traces of 40 neurons from UI and TI video before deconvolution. (c,d) Calcium traces of two neighboring neurons, corresponding to the gray shaded traces in (a,b). (e) Histogram of correlation coefficients of calcium traces between pairs of neurons in the UI (red) and TI (green) videos before deconvolution.

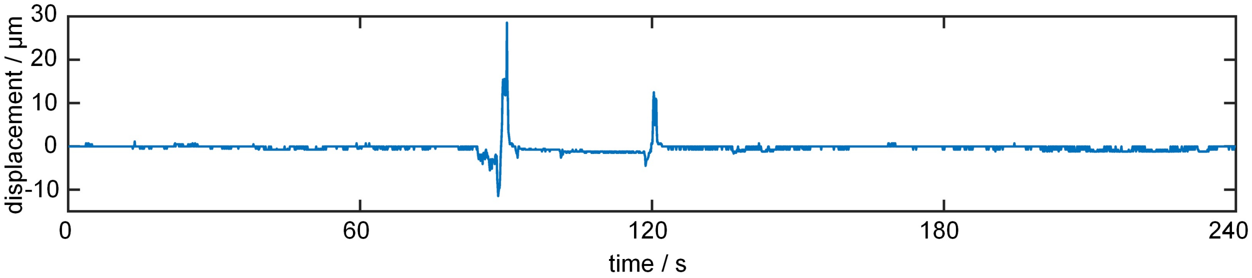

Figure 8. Brain motion during the 4 min acquisition time. The displacement was estimated by comparing every frame to the first frame of the video using band-limited phase-only correlation method. There are two major motion artifacts around 90 s and 120 s.
